# The restoration of REST inhibits reactivity of Down syndrome iPSC-derived astrocytes

**DOI:** 10.3389/fnmol.2025.1552819

**Published:** 2025-03-26

**Authors:** Tan Huang, Sharida Fakurazi, Pike-See Cheah, King-Hwa Ling

**Affiliations:** ^1^Department of Biomedical Sciences, Faculty of Medicine and Health Sciences, Universiti Putra Malaysia, Serdang, Selangor, Malaysia; ^2^Department of Human Anatomy, Faculty of Medicine and Health Sciences, Universiti Putra Malaysia, Serdang, Selangor, Malaysia; ^3^Malaysian Research Institute on Ageing (MyAgeing®), Universiti Putra Malaysia, Serdang, Selangor, Malaysia; ^4^Brain and Mental Health Research Advancement and Innovation Networks (PUTRA BRAIN), Universiti Putra Malaysia, Serdang, Selangor, Malaysia

**Keywords:** Down syndrome, iPSC, lithium, REST, reactive astocyte

## Abstract

**Introduction:**

Accumulating evidence indicates that the increased presence of astrocytes is fundamentally linked to the neurological dysfunctions observed in individuals with Down syndrome (DS). REST (RE1-silencing transcription factor), as a chromatin modifier, regulates 15,450 genes in humans. REST is a key regulatory element that governs astrocyte differentiation, development, and the maintenance of their physiological functions. The downregulation of REST may disrupt the homeostatic balance of astrocytes in DS.

**Methods:**

This study aims to elucidate the role of REST in DS-astrocytes through comprehensive transcriptomic analysis and experimental validation.

**Results:**

Transcriptomic analysis identified that REST-targeted differentially expressed genes (DEGs) in DS astrocytes are enriched in pathways associated with inflammatory response. Notably, our findings in astrocytes derived from DS human induced pluripotent stem cells (hiPSCs) show that the loss of nucleus REST leads to an upregulation of inflammatory mediators and markers indicative of the presence of reactive astrocytes. Lithium treatment, which restored nucleus REST in trisomic astrocytes, significantly suppressed the expression of these inflammatory mediators and reactive astrocyte markers.

**Discussion:**

These findings suggest that REST is pivotal in modulating astrocyte functionality and reactivity in DS. The loss of REST in DS-astrocytes prompts the formation of reactive astrocytes, thereby compromising central nervous system homeostasis. Lithium treatment possesses the potential to rescue astrocyte reactivity in DS by restoring nucleus REST expression.

## 1 Introduction

Down syndrome (DS) is the most common chromosomal genetic disorder associated with intellectual disability. The neurogenic-gliogenic shift could be a key neuropathological mechanism in DS (Huang et al., 2025). In the DS brain, not all glial cell lineages increase; astrocytes, particularly, are predominantly affected (Mizuno et al., 2018; Ponroy Bally and Murai, 2021). Although astrocyte differentiation increases during DS neurodevelopment, their development and maturation are impaired (Ponroy Bally and Murai, 2021). This leads to functional defects in astrocytes that disrupt their supportive effects on neurons and transform them into neurotoxic substances. DS astrocytes negatively affect synaptic development and maturation, neuronal ion channels, neuronal excitability and survivability (Ponroy Bally et al., 2020). RNA sequencing revealed that DS astrocytes undergo genome-wide transcriptional disruption, leaving DS neurodevelopment, cell adhesion, mitochondrial function, and extracellular matrix-related molecules severely dysregulated (Ponroy Bally et al., 2020; Kawatani et al., 2021). Notably, chromosome 21 genes are consistently upregulated in DS astrocytes, causing S100B to preferentially and significantly accumulate in the cells, leading to astrocyte dysfunction and oxidative stress-induced neural death and impaired neurogenesis (Royston et al., 1999; Lu et al., 2011; Chen et al., 2014). Nevertheless, this change does not affect DS astrocyte viability. However, there is limited information on the DS astrocyte characterisation.

Reactive astrocyte is a defensive response of astrocytes to pathological processes involving complex activation programmes that remodel astrocyte biochemical, morphological, metabolic, and physiological characteristics (Nakagawa et al., 2005; Hamby and Sofroniew, 2010; Ding et al., 2021). This can result in either neuroprotection or damage by upregulating or losing homeostatic cascades. Reactive astrocytes induced by neuroinflammation and/or injury can be categorised into “A1” and “A2” subtypes (Escartin et al., 2021). A1 reactive astrocytes upregulate proinflammatory factors such as *IL-1β*, *TNF-α*, and ROS and release cytotoxins, which are considered neurotoxic reactive astrocytes (Li et al., 2019). A2 reactive astrocytes upregulate the neurotrophic factors *STAT3*, *VEGF*, and *FGF-2*, as well as the anti-inflammatory factors *IL-10* and *TGF-β*, which are considered neuroprotective reactive astrocytes (Li et al., 2019). However, reactive astrocytes do not polarise into simple binary phenotypes and rarely does only a single phenotype exist in a disease. Acquisition of neuroprotective or neurotoxic function may occur simultaneously, depending on the balance of homeostasis in the disease environment (Liddelow and Barres, 2017; Escartin et al., 2021).

RE-1 silencing transcription factor (REST), or neuron-restrictive silencing factor (NRSF), is a crucial transcription factor regulating neurodevelopment, neuronal differentiation, and function maintenance (Hwang and Zukin, 2018; Alsaqati et al., 2022). REST recognises and binds to specific DNA sequences termed RE1 (Repressor Element 1), playing a central role in chromatin remodelling (Ballas et al., 2005). Estimations indicate the presence of approximately ~2,000 RE1 regulatory elements in the human genome, characterised by the known RE1 consensus sequence (Bruce et al., 2004; Johnson et al., 2006). Upon binding to RE1/NRSE sites, REST recruits various corepressors, including mSin3A and the CoREST complex (Maksour et al., 2020), as well as chromatin-modifying enzymes such as histone deacetylases and histone methyltransferases (Huang et al., 1999; Tahiliani et al., 2007). These interactions restructure the chromatin architecture, effectively blocking the access of transcriptional activators and RNA polymerase II, thereby silencing the transcription of target genes. The Gene Transcription Regulation Database (GTRD) has identified 15,450 REST target genes in the human genome (Kolmykov et al., 2021). Notably, ChIP-chip analysis revealed that the mapped RE1 binding sites across mouse-derived glial cells include 3,178 REST target genes and 4,060 CoREST target genes (Abrajano et al., 2009), underscoring the extensive regulatory network governed by REST.

Unlike neurons, REST is expressed at higher levels in astrocytes, vital in regulating astrocyte activities and disease-associated changes (Abrajano et al., 2009; Kohyama et al., 2010; Centonze et al., 2023). REST influences astrocyte functions, including glial reprogramming, glial lineage differentiation, vesicle trafficking, mitochondrial activity inflammation response, and synaptic plasticity regulation (Abrajano et al., 2009; Prada et al., 2011; Bergsland et al., 2014; Virmani et al., 2015). It remains unclear whether REST mediates the dysregulated molecular mechanism of DS-reactive astrocytes. This study aims to explore the reactivity of astrocytes derived from DS hiPSCs and investigate the impact of REST expression levels on astrocytic reactivity.

## 2 Materials and methods

### 2.1 Sequencing analysis

The high throughput sequencing dataset for DS hiPSC-derived astrocyte from the Sequence Read Archive database (SRA)^1^ was retrieved. The PRJNA600245 dataset included 12 DS samples and 12 matched isogenic controls (Ponroy Bally et al., 2020). The Galaxy web-based platform^2^ was used to analyse the Next-Generation Sequencing (NGS) dataset. The *Trim Galore* software (Galaxy Version 0.6.7 + galaxy0) was used to read and trim the FASTQ files. The *FASTQC* (Galaxy Version 0.74 + galaxy1) was used to test the quality of individual samples. Subsequently, the *HISAT2* software was employed to align the transcriptome sequences, using the *hg38* human reference genome for annotation. The *htseq-count* software (Galaxy Version 2.0.5 + galaxy0) was used to count aligned reads. Lastly, *DESeq2* software (Galaxy Version 2.11.40.8 + galaxy0) was utilised to analyse differentially expressed genes between DS and control groups. Genes were classified as DEGs based on adjusted *p*-value <0.05 (Benjamini-Hochberg method) and |log2 FC| > 1. The analysis results were visualised with a heatmap and volcano plot created using RStudio software (Version: 2023.03.0 + 386).

### 2.2 REST-targeted DEGs and their functions in DS-astrocytes

Human-specific REST target genes were retrieved from the Gene Transcription Regulation Database.^3^ To visualise the overlap compared these REST-targeted genes with the DS astrocyte DEGs from the PRJNA600245 dataset, Venn diagrams were generated using the web tool https://bioinfogp.cnb.csic.es/tools/venny/. The significance of the gene overlap was then evaluated using the hypergeometric probability model, with the human genome as the reference background.^4^

Gene Ontology (GO) (Ashburner et al., 2000) and Kyoto Encyclopedia of Genes and Genomes (KEGG) (Kanehisa et al., 2025) enrichment analyses were performed on DEGs and REST-targeted DEGs with R package *clusterProfiler* (Wu et al., 2021). The significant enrichment results from GO and KEGG with *p*-values <0.05 were considered. The *rrvgo* and *simplifyEnrichment* packages perform semantic similarity analysis on the GO analysis results and cluster the results. The *heatmapPlot* and *complexheatmap* packages were used to visualise the enriched GO and KEGG results.

### 2.3 Cell culture

#### 2.3.1 Production of neural progenitor cells (NPCs) from hiPSCs

Three trisomic iPSC lines [DS2 (WC-24-02-DS-M), DS3 (UWWC1-2DS3), and DS4 (HPS4270)] and three euploid iPSC lines [C2 (WC-24-02-DS-A), C4 (ATCC-DYS0100), and C5 (HPS4272)] were utilised in this study. These lines included two pairs of isogenic cell lines (DS2 and C2; DS4 and C5) and one pair of non-isogenic cell lines (DS3 and C4). DS2, C2, and DS3 were obtained from the WiCell Research Institute at the University of Wisconsin (Giffin-Rao et al., 2020; Weick et al., 2013); C5 and DS4 were sourced from the RIKEN BioResource Center in Japan (Wakita et al., 2022); and C4 was acquired from the American Type Culture Collection (Wren et al., 2015). Detailed information on the cell lines is provided in Table 1. HiPSCs were cultured in complete mTeSR™ Plus medium (STEMCELL Technologies, Canada, Cat. no. 100-0276) on Geltrex (Gibco, USA, Cat. no. A1413301) coated six-well plates, following the manufacturer’s instructions. Upon reaching approximately 80% confluency, the hiPSCs were dissociated with Accutase (Thermo Fisher, USA, Cat. no. 00-4555-56) and seeded onto Geltrex-coated six-well plates at a density of 300,000 cells per well in complete mTeSR™ Plus medium supplemented with 5 μM Y27632 (STEMCELL Technologies, Canada, Cat. no. 72304). For neural induction, hiPSCs at 20% confluency were exposed to a complete Neural Induction Medium (Gibco, USA, Cat. no. A1647801) for 7 days, with medium changes every 2 days, following the manufacturer’s instructions. Following the manufacturer’s instructions, the resulting hiPSC-derived NPCs were expanded in Neural Expansion Medium [comprising Neurobasal Medium, AdvancedTM DMEM/F-12 (Gibco, USA, Cat. no. 12634028)] on the Geltrex-coated 6-well plate.

**Table 1:** Details of the disomic control and trisomic DS iPSC lines.

Cell label	Cell line	Donor origin	Reprogramming method	Provider
C2	WC-24-02-DS-A	Mosaic DS individual Female Aged 25 Skin fibroblast	Non-integrating episomal vector	WiCell Research Institute, University of Wisconsin
DS2	WC-24-02-DS-M			
C4	ATCC-DYS0100	Healthy individual Male Neonate Skin fibroblast	Sendai viral transfection system	American Type Culture Collection (ATCC)
DS3	UWWC1-2DS3	Trisomy 21 individual Male Neonate Skin fibroblast	Non-integrating episomal vector	WiCell Research Institute, University of Wisconsin
C5	HPS4272	Trisomy 21 individual Male Aged <10 Skin fibroblast	Non-integrating episomal vector	RIKEN BioResource Center, Japan
DS4	HPS4270			

#### 2.3.2 Production of astrocytes from NPCs

NPCs were cultured at 60,000 cells per well in a 6-well plate coated with Geltrex. The cells were cultured in 2 mL of Neural Expansion Medium on the first day. The Neural Expansion Medium was replaced on the second day with 2 mL of complete Astrocyte Medium (Sciencell, USA; cat No. 1801). The Astrocyte Medium was changed every 2 days for 30 days. Cells were dissociated with Accutase and seeded onto a Geltrex-coated plate whenever the culture reached 100% confluency. During the first 30 days, the cell density per well was kept low to facilitate differentiation. After 30 days, the differentiated astrocytes were further expanded. When the cells reached 80% confluency, they were collected in 1.5 mL tubes or passed onto coverslips for subsequent experiments.

### 2.4 MTT assay

After dissociating the cells with Accutase, the cells were suspended in Neural Expansion Medium and seeded in 96-well plates at a density of 25,000 cells/cm^2^. The plate was incubated at 37°C with 5% CO_2_ for 12 h to allow the cells to adhere. After 12 h, an appropriate concentration of the test compound (lithium carbonate or X5050) was added, and the cells were cultured for an additional 24 h. Following this incubation period, the supernatant was carefully removed, and 90 μL of fresh medium was added to each well. Subsequently, 10 μL of MTT solution (Solarbio, China, Cat. no. M1020) was added, and the cells were incubated for another 4 h. Subsequently, the supernatant was discarded, and 110 μL of Formazan solubilisation solution was introduced into each well. The plate was then placed on a shaker and gently agitated for 10 min to ensure complete dissolution of the formazan crystals. Absorbance was subsequently assessed at 490 nm with a microplate reader. Blank wells (containing only medium, MTT, and Formazan solubilisation solution) and control wells (containing cells, the same concentration of drug solvent, medium, MTT, and Formazan solubilisation solution) were set up, with four replicates per group.

### 2.5 Quantitative real-time PCR

Quantitative real-time PCR was performed to evaluate gene expression levels following the manufacturer’s protocol. Total RNAs were extracted from astrocytes using the GeneAll® Ribospin™ II RNA extraction kit (Cat No. 314-150). RNA purity and concentration were measured with a NanoDrop™ 1000 spectrophotometer (Thermo Fisher Scientific). Reverse transcription was done using the LunaScript® RT SuperMix kit (New England Biolabs, USA, E3010). A total of 1 μg RNA was reverse-transcribed in a 20 μL reaction volume. Real-time quantitative RT-PCR was performed on the cDNA using LunaScript® qPCR Master Mix (New England Biolabs, USA, M3004), and the detection and analysis were conducted with the LightCycler® 480 Real-Time PCR System from Roche Diagnostics. Samples were prepared for duplicate assays. Relative gene expression between groups were analysed by using a standard curve-based relative quantification approach (Ling et al., 2009). A set of serially diluted cDNA samples (four data points) was used to construct standard curves for each PCR assay, with crossing points (Cp) determined by the second derivative maximum method to ensure accurate quantification (Luu-The et al., 2005). The expression levels were normalised to the geometric mean of two reference genes (*PSMB2* and *HMBS*) to account for variations in RNA input and reverse transcription efficiency. The primer sequences utilised for RT-qPCR are listed in Table 2.

**Table 2:** List of primer sequences for RT-qPCR.

Gene symbols	Forward primers (5′ → 3′)	GC%	Reverse primers (5′ → 3′)	GC%	Annealing temperature (°C)	Amplicon size (bp)
*GFAP*	CAGATTCGAGGGGGCAAA	55.56	TGAGAGGCAGGCAGCTA	58.82	60	149
*EAAT1*	ACATGAAGGAACAGGGGCAG	55.00	CACGGGGGCATACCACATTA	55.00	60	98
*EAAT2*	TGCCAACAGAGGACATCAGCCT	54.55	CAGCTCAGACTTGGAGAGGTGA	54.55	60	128
*HMBS*	GCCGTGCATACAGCTATGAAG	52.38	TTACGAGCAGTGATGCCTACC	52.38	60	179
*IL10*	GGCACCCAGTCTGAGAACAG	60.00	TGGCAACCCAGGTAACCCTTA	52.38	60	176
*IL1B*	GCTCTGGGATTCTCTTCAGCC	57.14	CAAGTCATCCTCATTGCCACTGT	47.83	60	118
*PSMB2*	GAGGGCAGTGGAACTCCTTAG	57.14	GATGTTAGGAGCCCTGTTTGG	52.38	60	149
*REST*	GAGAACGCCCATATAAATGTG	42.86	CACATAACTGCACTGATCAC	45.00	60	172
*S100A10*	CGCCGCACGTACTAAGGAA	57.89	GTGTGGTCCGTTGAAGCCTTG	57.14	60	118
*S100B*	GTGGCCCTCATCGACGTTTT	55.00	ACCTCCTGCTCTTTGATTTCCTCT	45.83	60	134
*TNF-α*	GCCCATGTTGTAGCAAACC	52.63	TCTGGTAGGAGACGGCGAT	57.89	60	226
*TGF-β*	TTGAGGGCTTTCGCCTTAGC	55.00	CGGTAGTGAACCCGTTGATG	55.00	60	84

F, Forward Primer; R, Reverse Primer; *GFAP*, Glial fibrillary acidic protein; *EAAT1*, Excitatory amino acid transporter 1; *EAAT2*, Excitatory amino acid transporter 2; *HMBS*, Hydroxymethylbilane synthase; *IL-10*, Interleukin 10; *IL-1B*, Interleukin-1 beta; *PSMB2*, Proteasome 20S subunit beta 2; *REST*, RE1 silencing transcription factor; *S100A10*, S100 calcium-binding protein A10; *S100B*, S100 calcium-binding protein B; *TNF-α*, Tumour necrosis factor; *TGF-β*, Transforming growth factor beta.

### 2.6 Immunocytochemistry

The astrocytes derived from hiPSCs were seeded onto Geltrex-coated glass coverslips in Neural Expansion Medium, with medium exchange occurring every second day. Subsequently, cells were fixed in 4% paraformaldehyde (PFA), rinsed with phosphate-buffered saline (PBS), permeabilised in a blocking solution containing 0.3% Triton X-100, 10% donkey serum, and PBS, and subjected to overnight staining at 4°C. The staining involved REST, EAAT2, and S100A10 antibodies in a mixture of 10% donkey serum, 0.3% Triton X-100, and PBS. Following PBS washes, secondary antibodies were applied for 2 h at room temperature, followed by nucleus counterstaining with DAPI. The primary antibodies employed for immunodetection were as follows: anti-REST (Host: Rabbit; Proteintech, USA; 1:200 dilution; Cat. no. 22242-1-AP), anti-OCT4 (Host: Rabbit; Sigma, USA, Cat. no. P0056; 1:200 dilution), anti-SOX2 (Host: Goat; Abcam, USA, Cat. no. ab239218; 1:200 dilution), anti-Nestin (Host: Mouse; Invitrogen, USA, Cat. no. MA1-110; 1:200 dilution), anti-GFAP (Host: Mouse; Invitrogen, USA, Cat. no. MA5-12023), anti-REST (Host: Rabbit; Proteintech, USA; 1:200 dilution; Cat. no. 22242-1-AP), anti-EAAT2 (Host: Rabbit; Invitrogen, USA; 1:200 dilution; Cat. no. 711020), anti-S100A10 (Host: Rabbit; Invitrogen, USA; 1:200 dilution; Cat. no. 6F4-E6-D5-C10), and anti-S100B (Host: mouse; Santa Cruz Biotechnology, USA; 1:100 dilution; Cat. no. sc-393919). The secondary antibodies employed for immunodetection were as follows: donkey anti-rabbit IgG (H+L) highly cross-adsorbed secondary antibody, Alexa Fluor™ 488 (Invitrogen, USA; 1:1000 dilution; Cat. no. A-21206) for OCT4, REST, EAAT2 and S100A10; donkey anti-mouse IgG (H+L) highly cross-adsorbed secondary antibody, Alexa Fluor™ 488 (Invitrogen, USA, A-21202; 1:1000 dilution) for NESTIN and GFAP; donkey anti-goat IgG H&L (Alexa Fluor® 594) (Abcam, USA, Cat. no. ab150132; 1:2000 dilution) for SOX2; donkey anti-mouse IgG H&L (Texas Red®) (Abcam, USA; 1:2000 dilution; Cat. no. ab6818) for S100B.

Image acquisition was performed using a fluorescence microscope (Olympus IX51, Japan). Fiji software^5^ was used to analyse the signal intensity of fluorescence images (Schindelin et al., 2012). For whole-cell fluorescence quantification, the total fluorescence intensity of the target protein across the entire image was measured. To account for variations in cell density, the total fluorescence intensity was normalised to the number of nuclei (DAPI positive) in the image. For the nucleus REST protein quantification, the astrocytes were stained with an anti-REST antibody and DAPI to label the nucleus. The nucleus region of each cell was delineated based on DAPI staining. The corresponding REST fluorescence intensity within the defined nucleus regions was measured. The nucleus REST fluorescence intensity of each nucleus was normalised by the average REST fluorescence per nucleus intensity of the control group (Con group).

### 2.7 Statistical analysis

The data are presented as mean ± standard deviation (SD), with all experiments conducted in triplicate. Statistical analyses were performed using GraphPad Prism 9.0 (version 9.5.0). An independent samples t-test was used to compare the differences between the two independent experimental groups. For comparisons involving three or more experimental groups, one-way ANOVA (Analysis of Variance) was employed to assess the differences between groups. A significance threshold of *p* < 0.05 was applied, and statistical significance is represented as follows: ns, not significant; *p < 0.05; **p < 0.01; ***p < 0.001.

## 3 Results

### 3.1 Transcriptomic profiling and functional implications of REST loss in DS hiPSC-derived astrocytes

**Figure 1:**
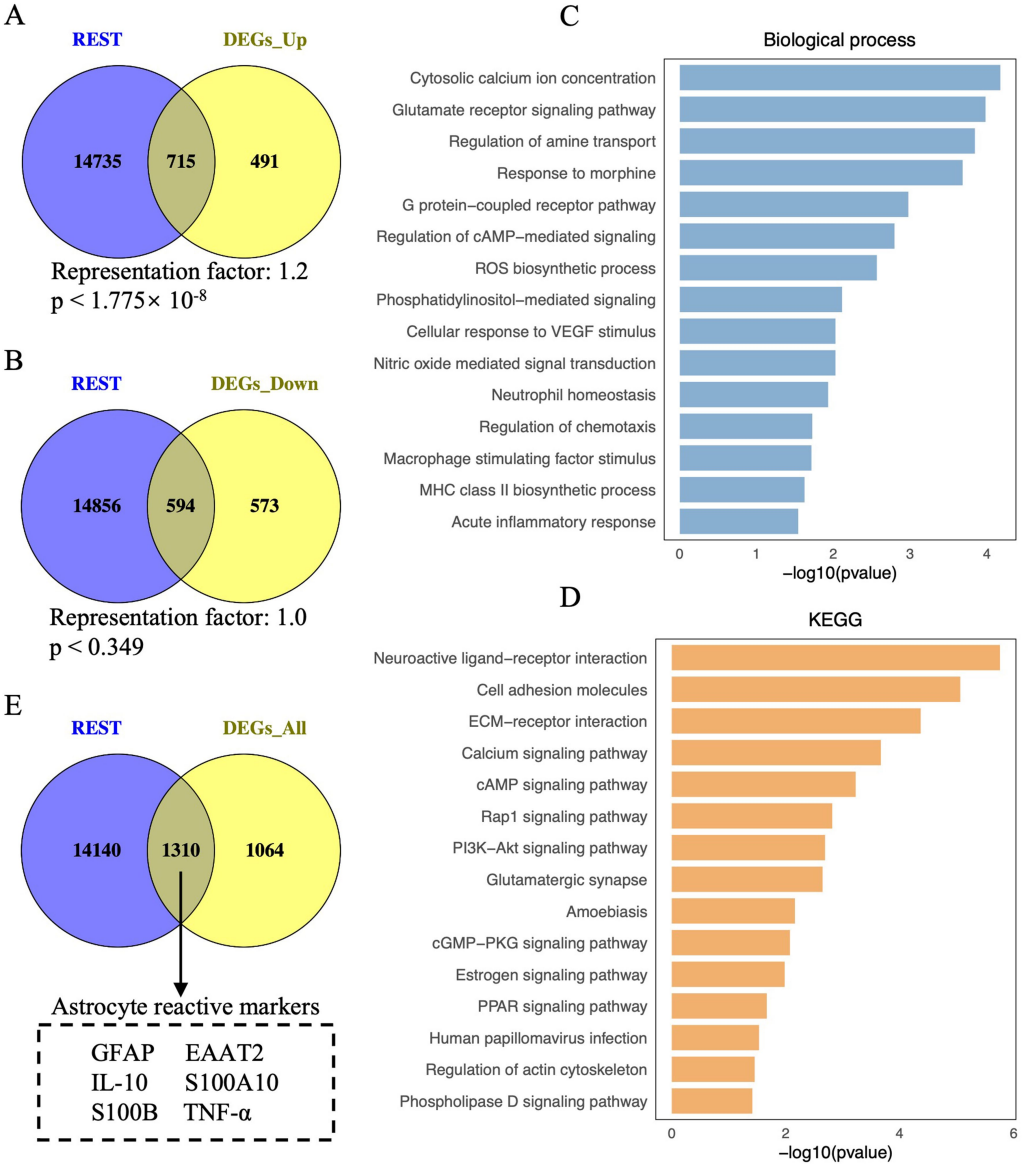
Visualisation of REST-targeted DEGs and enrichment analysis of REST-targeted upregulated DEGs in DS hiPSC-derived astrocytes. **(A)** Depiction of the overlap between REST target genes and the upregulated DEGs in DS iPSC-derived astrocytes. The hypergeometric test determined the statistical analysis of the gene overlap, and a *p*-value <0.01 is considered statistically significant. **(B)** Depiction of the overlap between REST target genes and the downregulated DEGs in DS iPSC-derived astrocytes. **(C)** Biological processes and **(D)** KEGG pathways for REST-targeted upregulated DEGs in DS iPSC-derived astrocytes. **(E)** Depiction of astrocyte reactive markers in the overlap between REST target genes and all DEGs (including upregulated and downregulated DEGs) in DS iPSC-derived astrocytes.

To comprehensively understand the molecular alterations in DS astrocytes, we analysed RNA sequencing data from the PRJNA600245 dataset (DS hiPSC-derived astrocytes). A total of 2,373 differentially expressed genes (DEGs) were identified, including 1,206 upregulated and 1,167 downregulated genes (Figures 1A,B). Heatmap and volcano plot visualisations are shown in Supplementary Figure S1.

To explore the role of REST in these transcriptional changes, we analysed the overlap between REST target genes and the DEGs. A significant overlap was observed between REST target genes and upregulated DEGs, with 715 REST-targeted DEGs identified (representation factor, RF: 1.2; *p* < 1.775 × 10^−8^) (Figure 1A). Notably, these included key genes associated with astrocyte reactivity and inflammation, such as GFAP, S100B, and TNF-α. In contrast, the overlap between REST target genes and downregulated DEGs was not statistically significant, with 594 REST-targeted DEGs identified (RF: 1.0; *p* = 0.349) (Figure 1B). These findings suggest a loss of REST function in DS astrocytes, consistent with its role as a transcriptional repressor.

To further investigate the functional implications of REST loss, we performed GO and KEGG enrichment analyses on the REST-targeted upregulated DEGs (Figures 1C,D). GO analysis revealed significant enrichment in biological processes related to inflammation and astrocyte reactivity, including cytosolic calcium ion concentration, glutamate receptor signalling, ROS biosynthesis, nitric oxide-mediated signal transduction, neutrophil homeostasis, regulation of chemotaxis, and acute inflammatory response (Figure 1C). KEGG pathway analysis highlighted enrichment in neuroactive ligand-receptor interaction, ECM-receptor interaction, calcium signalling, Rap1 signalling, PI3K-Akt signalling, amoebiasis, cGMP-PKG signalling, and PPAR signalling pathways (Figure 1D). These results indicate that REST loss disrupts astrocyte homeostasis and promotes inflammatory signalling.

Importantly, several key genes associated with reactive astrocytes, such as *GFAP*, *EAAT2*, *IL-10*, *S100A10*, *S100B*, and *TNF-α*, were identified among the REST-targeted DEGs (Figure 1E). This finding underscores the potential role of REST in modulating reactive astrocyte phenotypes and suggests that REST loss contributes to the inflammatory and reactive state of DS astrocytes.

### 3.2 Astrocyte differentiation and characterisation

**Figure 2:**
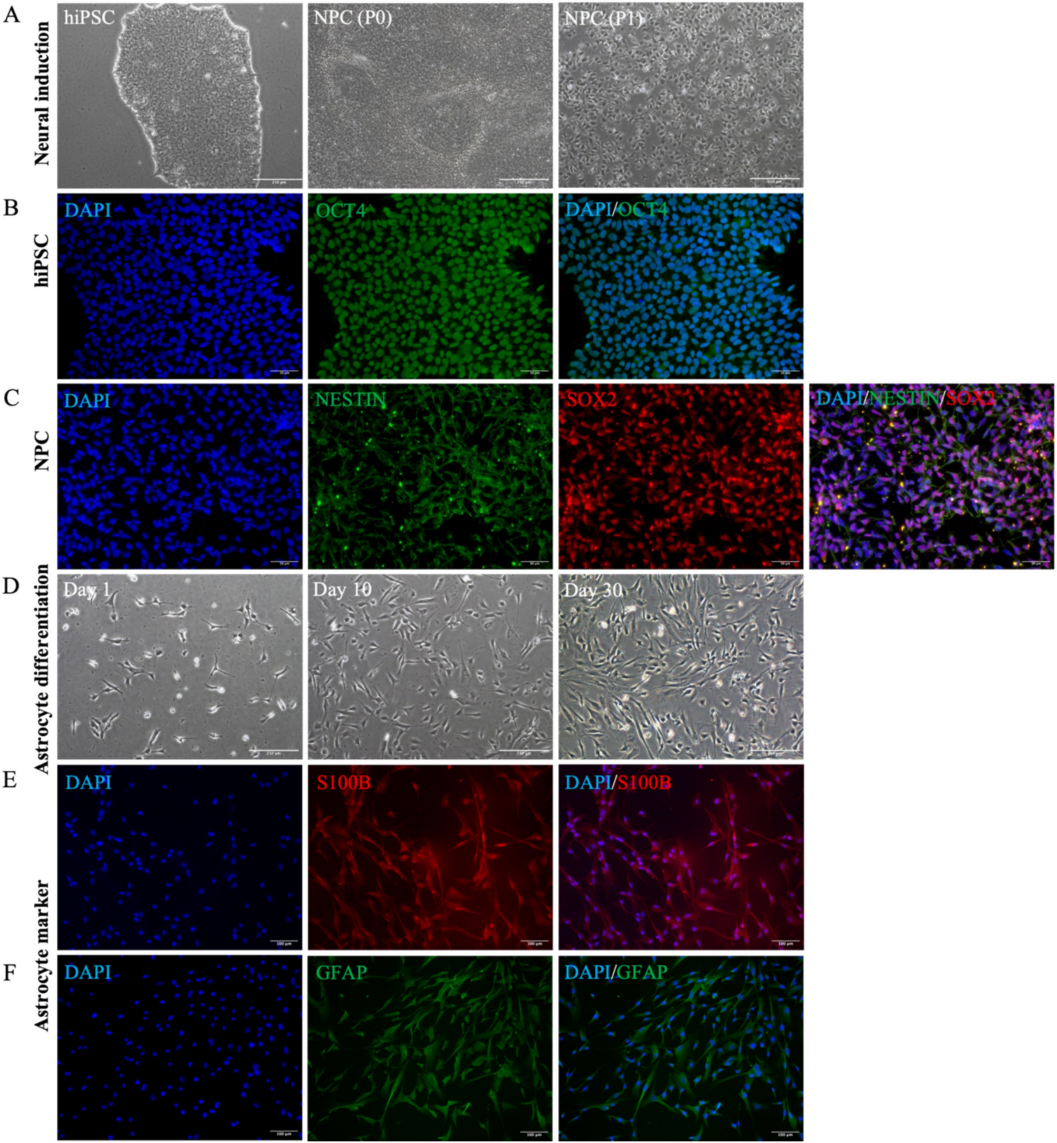
Astrocyte differentiation and identification of cellular characteristics. **(A)** The cellular morphological characteristics of hiPSCs, P0 NPCs, and P1 NPCs during the cultivation process (scale bar = 250 μm). **(B)** HiPSCs were identified by OCT4 cell marker (scale bar = 50 μm). **(C)** All NPCs expressed Nestin and SOX2 cell markers (scale bar = 50 μm). **(D)** The cell morphological changes were observed during this 30-day astrocyte differentiation period. Bright-field microscopy images of astrocytes at day 1, day 10, and day 30 during differentiation (scale bar = 250 μm). **(E)** All astrocytes were positive for S100B expression (scale bar = 100 μm). **(F)** All astrocytes were positive for GFAP expression (scale bar = 100 μm).

To investigate the role of REST in DS astrocytes, we differentiated hiPSCs into astrocytes and characterised the intermediate and final cell populations. Initially, hiPSCs were expanded, and their pluripotency was confirmed through immunocytochemical staining for the pluripotency marker OCT4 (Figures 2A,B). Subsequently, hiPSCs were subjected to neural induction using PSC Neural Induction Medium for 7 days, resulting in the generation of neural progenitor cells (NPCs), designated as passage P0 NPCs (Figure 2A). To ensure the stability and homogeneity of the NPC population, cells were expanded for at least one passage and characterised using immunocytochemistry. The NPCs uniformly expressed the neural progenitor markers Nestin and SOX2, confirming their identity (Figure 2C). Following 30 days of culture in Astrocyte Medium, NPCs successfully differentiated into astrocytes (Figure 2D). The astrocytic identity of the differentiated cells was validated through immunocytochemical staining for the mature astrocyte markers S100B and GFAP (Figures 2E,F). These results demonstrate the successful generation of hiPSC-derived astrocytes, providing a robust model for further investigation of REST function in astrocyte biology.

### 3.3 Loss of REST in DS-astrocytes restored by lithium treatment

**Figure 3:**
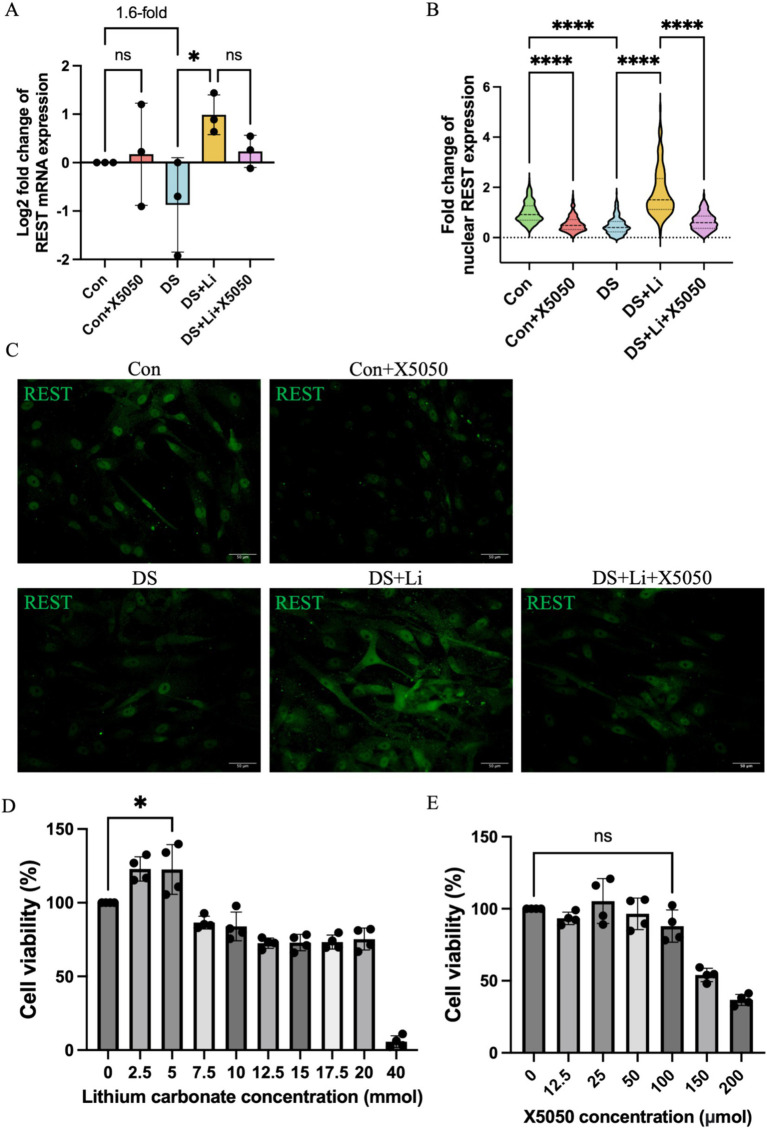
The detection of REST mRNA and protein in treated and untreated DS hiPSC-derived astrocytes and controls. Three pairs of cell lines (C2 vs. DS2, C4 vs. DS3, and C5 vs. DS4) were used to perform ICC. **(A)** The mRNA expression levels of *REST* in different groups: Con group (Control; Disomic astrocytes), Con + X5050 group (Disomic astrocytes with X5050 treatment), DS + Li group (Trisomic astrocytes with lithium carbonate treatment), DS + Li + X5050 (Trisomic astrocytes treated with both lithium carbonate and X5050). The mRNA expression levels were normalised to two reference genes (HMBS and PSMB2), and the relative mRNA expression levels for each group were calculated. The relative expression levels of each group were normalised to those of the Con group to obtain log2 fold changes for statistical analyses. **(B)** Nucleus REST expression analysis based on the **(C)** fluorescence micrographs. **(D)** Optimisation of safety dosage of lithium treatment in iPSC-derived astrocytes through MTT cell viability assay. Treatment with lithium carbonate up to 5 mM for 24 h is considered safe for astrocytes in which the cell viability does not drop below 100%. **(E)** Optimising safe doses of lithium for iPSC-derived astrocytes via MTT cell viability assay. Cell viability treated with 100 μM X5050 for 24 h was not statistically significant compared to the control and was considered safe. The one-way ANOVA was employed to assess the differences between groups. ns, not significant; *p < 0.05; **p < 0.01; ***p < 0.001, ****p < 0.0001. Scale bar = 50 μm.

To investigate the role of REST in DS hiPSC-derived astrocytes, we first compared REST expression levels between DS and control astrocytes (Figures 3A,B). Quantitative analysis revealed that *REST* mRNA levels in DS astrocytes were downregulated by 1.6-fold compared to the control group, although this difference was not statistically significant (Figure 3A). Given the critical role of REST as a nucleus transcription factor, we further assessed its protein levels in the nucleus. Immunocytochemical analysis demonstrated a significant reduction in nucleus REST protein levels in DS astrocytes relative to controls (*p* < 0.0001, Figures 3B,C).

To determine whether REST expression could be restored, DS astrocytes were treated with lithium carbonate. An MTT assay was performed to identify the optimal concentration of lithium carbonate for treatment. The results indicated that 5 mM lithium carbonate treatment for 24 h yielded the highest relative cell viability (122.5%) without compromising cell health (Figure 3D). Following treatment with 5 mM lithium carbonate (DS + Li group), REST mRNA levels were significantly upregulated by 3.3-fold compared to untreated DS astrocytes (*p* < 0.05; Figure 3A). Consistent with this, immunocytochemistry revealed a marked increase in nucleus REST protein levels in the DS + Li group compared to the DS group (*p* < 0.0001; Figures 3B,C).

To further validate the specificity of the effects of lithium on REST, we treated control astrocytes with X5050, a compound known to degrade REST protein. The MTT assay confirmed that 100 μM X5050 was a safe and effective concentration for 24-h treatment, as it did not significantly affect cell viability (Figure 3E). In control astrocytes treated with 100 μM X5050 (Con + X5050 group), *REST* mRNA levels remained unchanged compared to the control group. However, immunocytochemistry showed a significant reduction in nucleus REST protein levels in the Con + X5050 group (*p* < 0.0001; Figures 3B,C). Importantly, when DS astrocytes were co-treated with 5 mM lithium carbonate and 100 μM X5050 (DS + Li + X5050 group), X5050 effectively counteracted the lithium-induced upregulation of nucleus REST protein (Figures 3B,C).

### 3.4 Restoration of REST downregulated the REST-targeted inflammatory genes

**Figure 4:**
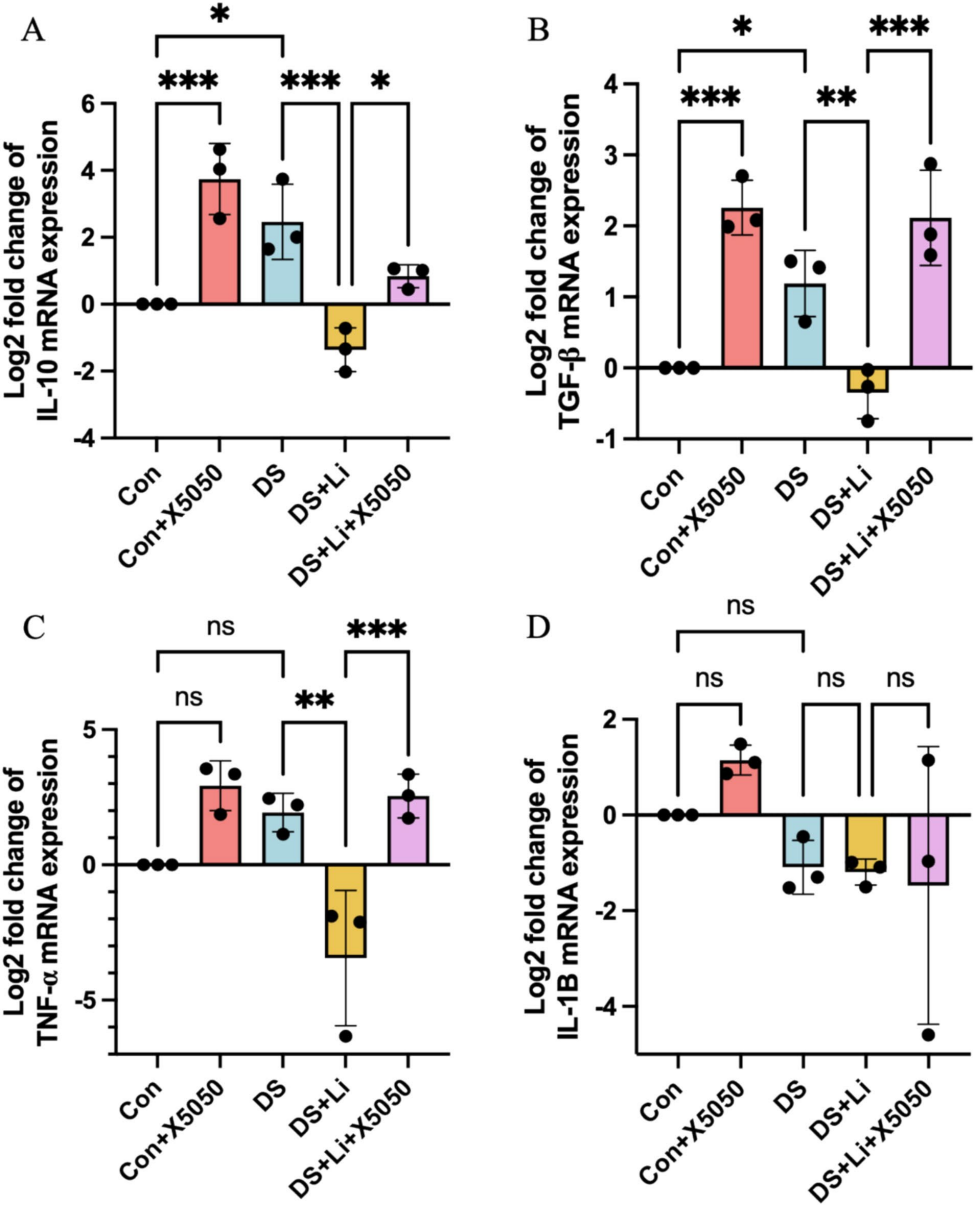
The detection of inflammation factors in treated and untreated DS hiPSC-derived astrocytes and controls. Three pairs of cell lines (C2 vs. DS2, C4 vs. DS3, and C5 vs. DS4) were used to perform RT-qPCR. The mRNA expression levels of inflammatory cytokines *IL-10* **(A)**, *TGF-β* **(B)**, *TNF-α* **(C)**, and *IL-1B* **(D)** were analysed across different groups: Con group (Disomic astrocytes), Con + X5050 group (Disomic astrocytes with X5050 treatment), DS + Li group (Trisomic astrocytes with lithium carbonate treatment for 24 h), DS + Li + X5050 (Trisomic astrocytes with both lithium carbonate and X5050 treatment for 24 h). The mRNA expression levels were normalised to two reference genes (HMBS and PSMB2), and the relative mRNA expression levels for each group were calculated. The relative expression levels of each group were normalised to those of the Con group to obtain log2 fold changes for statistical analyses. The one-way ANOVA was employed to assess the differences between groups. ns, not significant; *p < 0.05; **p < 0.01; ***p < 0.001.

Neuroinflammation is a hallmark of DS that persists throughout the lifespan, with varying patterns across different life stages. To investigate the impact of REST loss on inflammatory responses in astrocytes, we quantified the mRNA expression levels of key inflammatory cytokines, including *IL-10*, *IL-1B*, *TNFα*, and *TGF-β*, in DS hiPSC-derived astrocytes (DS group) and controls (Con group) (Figure 4). These cytokines were selected based on their roles in regulating proinflammatory and anti-inflammatory signalling pathways, as well as their potential involvement in astrocyte activation (Hamby and Sofroniew, 2010; Liddelow and Barres, 2017).

In DS astrocytes, *IL-10* and *TGF-β* expression levels were significantly upregulated by 2.4-fold and 2.8-fold, respectively, compared to the control group (*p* < 0.05; Figures 4A,B). In contrast, *TNFα* and *IL-1B* expression levels did not show statistically significant changes, while *IL-1B* was downregulated by 2.0-fold and TNFα was upregulated by 4.1-fold (Figures 4C,D). To determine whether these changes were directly linked to the loss of nucleus REST, we treated control astrocytes with X5050, a compound that degrades REST protein (Con + X5050 group). Similar to DS astrocytes, X5050-treated astrocytes exhibited significant upregulation of *IL-10* (*p* < 0.001) and *TGF-β* (*p* < 0.001) compared to untreated controls (Figures 4A,B), while IL-1B and *TNFα* expression levels remained unchanged (Figures 4C,D). These results suggest that REST loss is a key driver of inflammatory dysregulation in DS astrocytes.

Next, we examined whether restoring REST expression through lithium carbonate treatment (DS + Li group) could reverse these inflammatory changes. Compared to untreated DS astrocytes, the DS + Li group showed significant downregulation of *IL-10* (*p* < 0.01), *TNFα* (*p* < 0.01), and *TGF-β* (*p* < 0.01) (Figures 4A–C), but not *IL-1B* (Figure 4D). Importantly, when DS astrocytes were co-treated with lithium carbonate and X5050 (DS + Li + X5050 group), the upregulation of *TGF-β* (*p* < 0.05), *IL-10* (*p* < 0.01), *TNFα* (*p* < 0.001), and *TGF-β* (*p* < 0.001) was restored, indicating that X5050 counteracted the anti-inflammatory effects of lithium treatment (Figures 4A–C). Notably, *IL-1B* expression did not correlate with REST levels, suggesting that REST may exert differential regulatory effects on specific inflammatory genes depending on its cellular concentration. These findings demonstrate that REST loss contributes to the dysregulation of inflammatory pathways in DS astrocytes and that REST restoration can effectively rescue these changes, highlighting its potential as a therapeutic target.

### 3.5 Restoration of REST suppresses the expression of markers for reactive astrocytes

**Figure 5:**
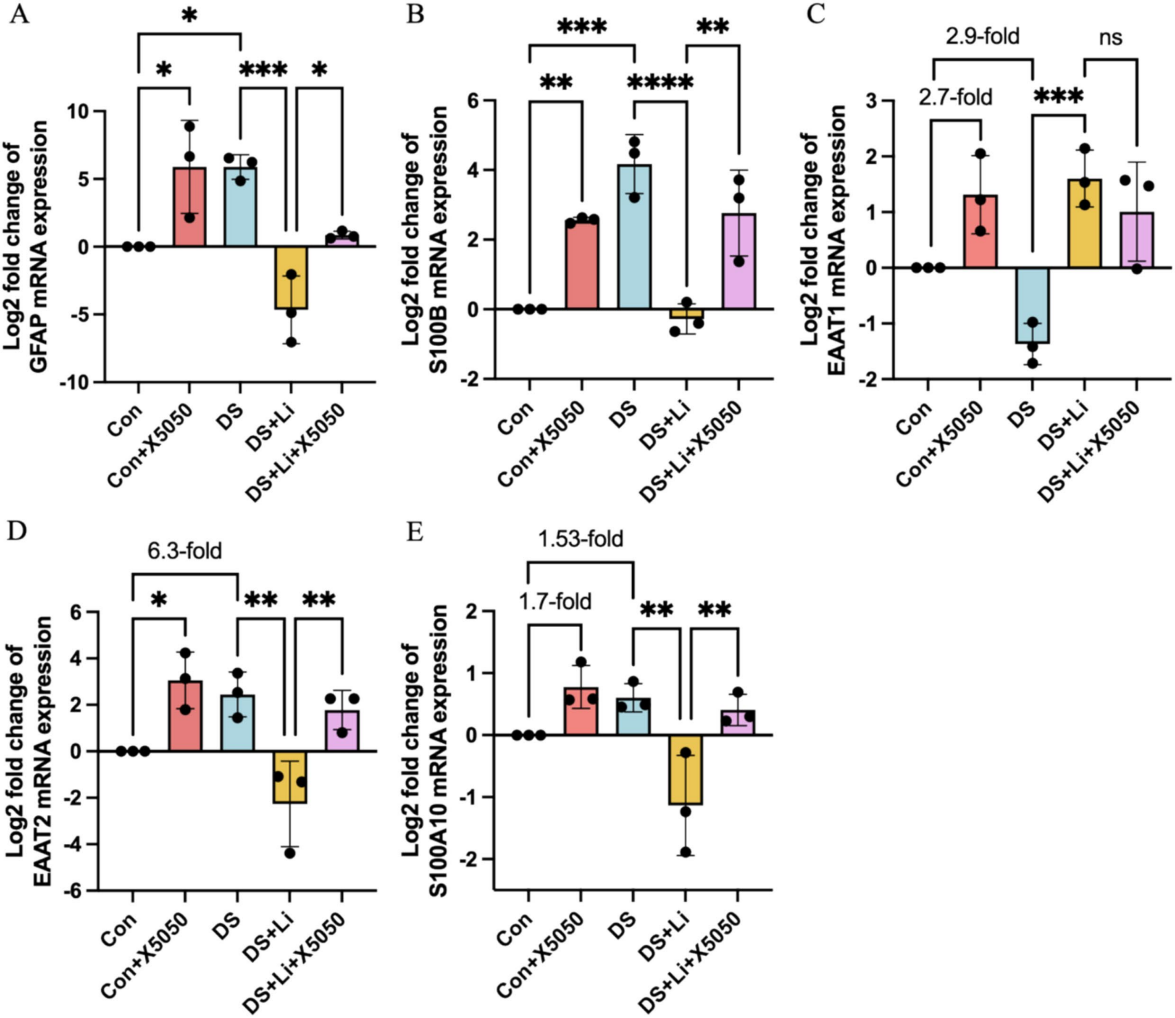
The mRNA expression of reactive astrocyte cell markers in treated and untreated DS hiPSC-derived astrocytes and controls. Three pairs of cell lines (C2 vs. DS2, C4 vs. DS3, and C5 vs. DS4) were used to perform RT-qPCR. The mRNA expression levels of reactive astrocyte cell markers *GFAP* **(A)**, *S100B* **(B)**, *EAAT1* **(C)**, *EAAT2* **(D)** and *S100A10* **(E)** were analysed across different groups: Con group (Control; Disomic astrocytes), Con + X5050 group (Disomic astrocytes with X5050 treatment), DS + Li group (Trisomic astrocytes with lithium carbonate treatment), DS + Li + X5050 (Trisomic astrocytes with both lithium carbonate and X5050 treatment). The mRNA expression levels were normalised to two reference genes (HMBS and PSMB2), and the relative mRNA expression levels for each group were calculated. The relative expression levels of each group were normalised to those of the Con group to obtain log2 fold changes for statistical analyses. The resulting values were then log-transformed (base 2) for standardisation, and the results were analysed statistically. The one-way ANOVA was employed to assess the differences between groups. ns, not significant; *p < 0.05; **p < 0.01; ***p < 0.001, ****p < 0.0001.

**Figure 6:**
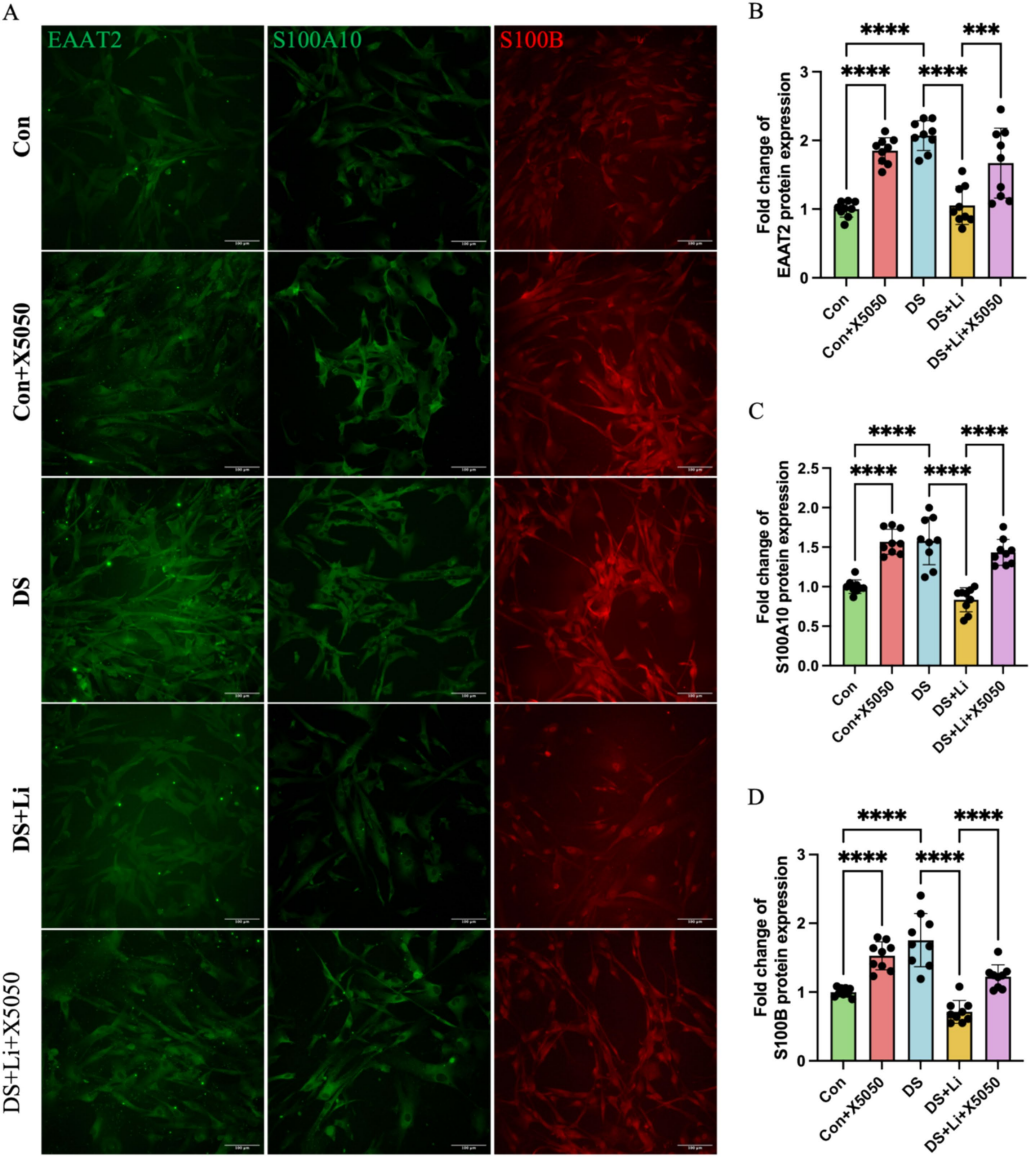
Immunocytochemical detection of DS hiPSC-derived reactive astrocyte markers. Three pairs of cell lines (C2 vs. DS2, C4 vs. DS3, and C5 vs. DS4) were used to perform ICC. Fluorescence micrographs of EAAT2, S100A10 and S100B staining and statistical analysis across different groups: Con group (Disomic astrocytes), Con + X5050 group (Disomic astrocytes with X5050 treatment), DS + Li group (Trisomic astrocytes with lithium carbonate treatment), DS + Li + X5050 (Trisomic astrocytes with both lithium carbonate and X5050 treatment). The one-way ANOVA was employed to assess the differences between groups. ns, not significant; *p-value < 0.05; **p-value < 0.01; ***p-value < 0.001, ****p-value < 0.0001. Scale bar = 100 μm.

Astrocytes can transition from a resting state to a reactive state in response to inflammatory mediators, characterised by the upregulation of specific markers associated with astrocyte activation (Escartin et al., 2021). To investigate the relationship between the loss of nucleus REST and reactive astrocyte formation, we analysed the mRNA and protein expression levels of key reactive astrocyte markers, including EAAT1, EAAT2, GFAP, S100A10, and S100B, in DS hiPSC-derived astrocytes (DS group) and controls (Con group) (Figures 5, 6). These markers were selected based on their roles in astrocyte reactivity and glutamate homeostasis, with EAAT1 and EAAT2 being critical indicators of glutamate-clearing capacity (Escartin et al., 2021; Pajarillo et al., 2021).

In DS astrocytes, *GFAP* and *S100B* mRNA expression levels were significantly elevated compared to controls, with *p* < 0.05 and *p* < 0.001, respectively (Figures 5A,B). In contrast, the expression levels of *EAAT1*, *EAAT2*, and *S100A10* did not show statistically significant differences, while *EAAT2* was increased by 6.3-fold and S100A10 was increased by 1.53-fold (Figures 5C–E). To determine whether these changes were directly linked to REST loss, we treated control astrocytes with X5050, a compound that degrades REST protein (Con + X5050 group). Similar to DS astrocytes, X5050-treated astrocytes exhibited significant upregulation of *EAAT2* (*p* < 0.05), *GFAP* (*p* < 0.05) and *S100B* (*p* < 0.001), while *EAAT1* and *S100A10* expression levels were elevated by 2.7-fold and 1.7-fold, respectively (Figures 5C,D). These results suggest that REST loss is a key driver of reactive astrocyte marker dysregulation.

Next, we examined whether REST restoration through lithium carbonate treatment (DS + Li group) could reverse these changes. Compared to untreated DS astrocytes, the DS + Li group showed significant downregulation of *GFAP* (*p* < 0.001), *S100B* (*p* < 0.0001), *EAAT2* (*p* < 0.01), and *S100A10* (*p* < 0.01), while *EAAT1* expression was elevated (Figure 5A,B,D,E). To confirm the specificity of the role of REST, we co-treated DS astrocytes with lithium carbonate and X5050 (DS + Li + X5050 group). In this group, the upregulation of *GFAP*, *S100B*, *EAAT2*, and *S100A10*, was restored, indicating that X5050 counteracts the effects of REST restoration (Figure 5A,B,D,E).

Immunocytochemical analysis further validated these findings at the protein level. In DS astrocytes, the protein levels of EAAT2, S100A10, and S100B were significantly upregulated compared to controls (*p* < 0.0001; Figure 6). Similarly, X5050-treated control astrocytes (Con + X5050 group) exhibited increased protein expression of these markers, further supporting the link between REST loss and reactive astrocyte formation (*p* < 0.001; Figure 6). In the DS + Li group, the protein levels of EAAT2, S100A10, and S100B were significantly reduced compared to untreated DS astrocytes (*p* < 0.0001; Figure 6). However, in the DS + Li + X5050 group, X5050 counteracts the effects of REST restoration, leading to a notable increase in protein levels of these markers (*p* < 0.001; Figure 6). Collectively, these results demonstrate that REST loss contributes to the upregulation of reactive astrocyte markers in DS hiPSC-derived astrocytes and that REST restoration can effectively suppress these changes. These findings highlight the potential of REST as a therapeutic target for modulating astrocyte reactivity in DS.

## 4 Discussion

Reactive astrocytes are central to neuropathological processes, exhibiting altered functions in neurotrophic support, neurotransmitter regulation, and inflammatory responses. In DS, astrocyte dysfunction is increasingly recognised as a key contributor to neurodevelopmental and neurodegenerative pathologies. Our study provides novel insights into the role of the transcriptional repressor REST in driving astrocyte reactivity and inflammatory dysregulation in DS. By integrating transcriptomic profiling, functional assays, and pharmacological interventions, we demonstrate that REST deficiency in DS hiPSC-derived astrocytes leads to upregulating inflammatory genes and reactive astrocyte markers, while REST restoration mitigates these effects. These findings elucidate the molecular mechanisms underlying astrocyte dysfunction in DS and highlight REST as a potential therapeutic target.

Our transcriptomic analysis identified REST-targeted differentially expressed genes (DEGs) associated with astrocyte reactivity, including GFAP, S100B, EAAT2, IL-10, and TNF-α. These findings align with previous studies implicating REST in regulating astrocyte development, inflammation, and synaptic function (Pajarillo et al., 2021; Huang et al., 2023). REST is known to repress genes involved in inflammation and oxidative stress, and its loss has been linked to neurodegenerative diseases such as Alzheimer’s disease and Parkinson’s disease (Lu et al., 2014; Buffolo et al., 2021). In DS, the downregulation of nucleus REST in astrocytes correlates with elevated expression of reactive astrocyte markers and inflammatory cytokines, suggesting that REST loss disrupts astrocyte homeostasis and promotes a reactive phenotype. Notably, lithium treatment restored nucleus REST levels and suppressed the expression of these markers, while X5050-mediated REST degradation reversed these effects, further underscoring the critical role of REST in modulating astrocyte reactivity.

The loss of REST in DS astrocytes resulted in the upregulation of key inflammatory mediators, including IL-10, TNF-α, and TGF-β, which are known REST targets (Abrajano et al., 2009; Kong et al., 2016). This inflammatory cascade was recapitulated in control astrocytes treated with X5050, confirming that REST deficiency is a key driver of inflammatory dysregulation. Interestingly, IL-1B expression was not directly correlated with REST levels, consistent with findings in Parkinson’s disease models (Pajarillo et al., 2021), suggesting that alternative pathways may regulate IL-1B. Recent studies have shown that IL-1B can induce REST expression, creating a feedback loop that modulates inflammatory responses (Buffolo et al., 2021). This may explain the complex relationship between REST and IL-1B in DS astrocytes. Additionally, REST has been shown to regulate the TGF-β pathway, which plays a critical role in astrocyte activation and neuroinflammation (Kong et al., 2016). The upregulation of both proinflammatory (TNF-α) and anti-inflammatory (TGF-β and IL-10) cytokines highlights the dual role of REST in balancing astrocyte-mediated inflammatory responses.

Our findings reveal a dual role for reactive astrocytes in DS, characterised by the coexistence of neurotoxic and neuroprotective phenotypes. The upregulation of GFAP and S100B, markers of neurotoxic astrocytes, alongside EAAT2 and S100A10, markers of neuroprotective astrocytes, reflects the multifaceted nature of astrocyte reactivity in DS (Liddelow and Barres, 2017; Escartin et al., 2021). This duality may underlie the progressive neuropathology observed in DS, where chronic inflammation and oxidative stress contribute to neuronal dysfunction and cell death (Chen et al., 2014). EAAT1 and EAAT2, considered protective markers, prevent synaptic glutamate overflow and minimise overactivation of extrasynaptic receptors, protecting against excitotoxicity (Price et al., 2021). In this study, we found no significant correlation between EAAT1 expression in DS-derived astrocytes and REST levels, consistent with findings in PD-derived astrocytes, which showed that REST does not influence EAAT1 transcription (Pajarillo et al., 2021). Recent studies have shown that neurotoxic astrocytes can exacerbate neurodegeneration by releasing proinflammatory cytokines and reactive oxygen species, while neuroprotective astrocytes promote neuronal survival through the release of neurotrophic factors and glutamate clearance (Liddelow and Barres, 2017; Escartin et al., 2021). The restoration of REST levels via lithium treatment downregulated both neurotoxic and neuroprotective markers, suggesting that REST acts as a master regulator of astrocyte reactivity.

Our study highlights the potential of REST as a therapeutic target for managing astrocyte-related neuropathology in DS. By restoring REST levels, we could suppress inflammatory gene expression and reactive astrocyte markers, offering a promising strategy to mitigate astrocyte-mediated neuroinflammation and neuronal dysfunction. Lithium, a well-known mood stabiliser, has been shown to enhance REST expression and function, making it a potential candidate for therapeutic intervention (Huang et al., 2023, 2025). However, the complexity of astrocyte reactivity and the dual roles of reactive astrocytes necessitate further investigation into the mechanisms underlying REST-mediated regulation of astrocyte functions. Future studies should explore the *in vivo* effects of REST modulation on astrocyte function and neuronal networks, as well as the potential of REST-targeted therapies for DS and other neurodevelopmental disorders. Additionally, developing small molecules or gene-editing technologies to specifically modulate REST expression in astrocytes could provide new avenues for therapeutic intervention.

## 5 Conclusion

In conclusion, our study demonstrates that REST deficiency in DS hiPSC-derived astrocytes drives inflammatory dysregulation and astrocyte reactivity, contributing to the neuropathology of Down syndrome. The loss of REST leads to the upregulation of inflammatory genes and reactive astrocyte markers, while REST restoration effectively suppresses these changes, highlighting its potential as a therapeutic target. Our findings underscore the dual role of reactive astrocytes in DS, characterised by both neurotoxic and neuroprotective phenotypes, and provide new insights into the molecular mechanisms underlying astrocyte dysfunction in DS. Future research should explore the in vivo effects of REST modulation on astrocyte function and neuronal networks, as well as the potential of REST-targeted therapies for DS and other neurodevelopmental disorders.

## Data Availability

The original contributions presented in the study are included in the article/Supplementary material, further inquiries can be directed to the corresponding author.

## Footnotes

^1^ https://www.ncbi.nlm.nih.gov/sra

^2^ https://www.usegalaxy.org/

^3^ http://gtrd.biouml.org

^4^ http://nemates.org

^5^ https://imagej.net/software/fiji/
