# Supplementary material for: The restoration of REST inhibits reactivity of Down syndrome iPSC-derived astrocytes
Source: Front Mol Neurosci. 2025 Mar 26;18:1552819. doi: 10.3389/fnmol.2025.1552819 (PMC11979110; doi:10.3389/fnmol.2025.1552819)
Supplement: Supplementary file 1 [file Supplementary_file_1.pdf]

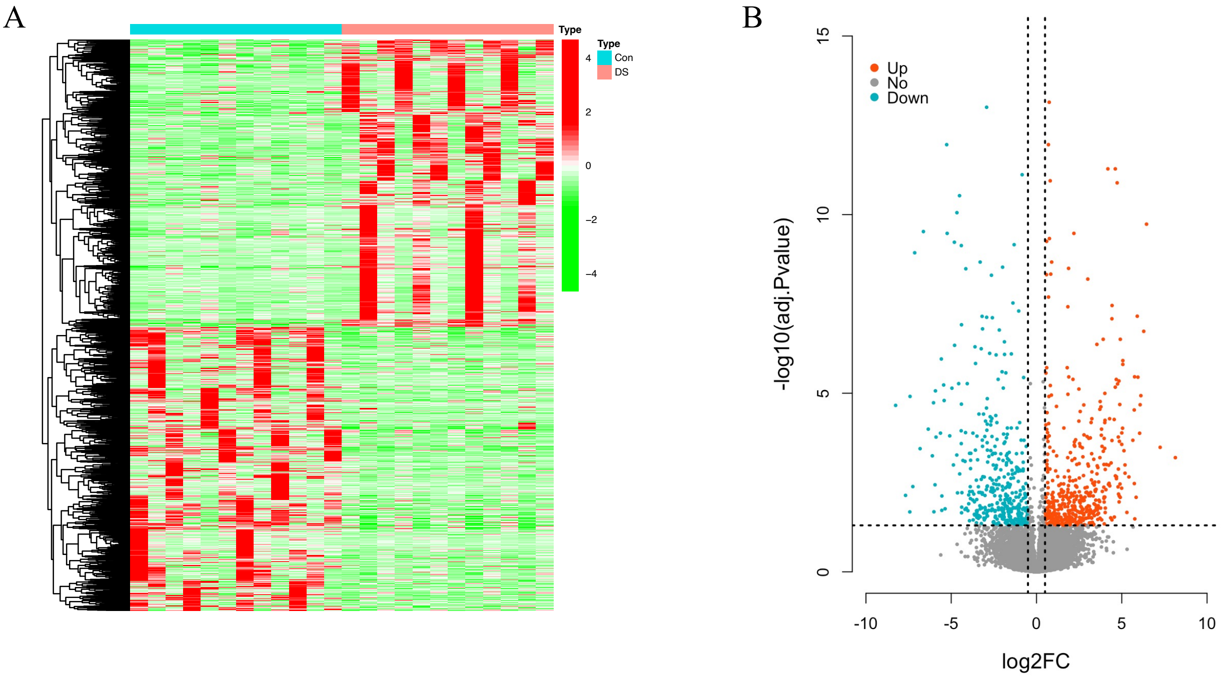

Figure S1. The visualization of identified differentially expressed genes in DS hiPSC-derived astrocytes. (A) Heatmap of DEGs DS iPSC-derived astrocytes; Red represents upregulated genes, while green represents downregulated genes. (B) Volcano map of DEGs DS iPSC-derived astrocytes; Red represents upregulated genes, green and blue represents downregulated genes and grey represents genes with no differential expression.
